# Multiple floods interactions shape riparian plant communities and diversity

**DOI:** 10.1038/s41598-025-05938-6

**Published:** 2025-07-02

**Authors:** Kota Igarashi, Takeshi Osawa

**Affiliations:** 1 Graduate School of Urban Environmental Sciences, Tokyo Metropolitan University, Minami-Osawa 1-1, Hachiouji, Tokyo 192-0397 Japan; 2 Present Address: Yachiyo Engineering Co., Ltd., Asakusabashi 5-20-8, Taito-ward, Tokyo 111-8648 Japan

**Keywords:** Disturbance interaction, Flooding, Plant community, Riparian ecosystem, Typhoon Hagibis, Biodiversity, Community ecology, Riparian ecology, Ecology, Freshwater ecology

## Abstract

Disturbances are pivotal ecological events that influence the formation of biological communities. Although disturbances have been studied in isolation, studies recently have highlighted the interaction among multiple disturbances. Riparian plant communities are primarily shaped by flooding disturbances, where periodic seasonal floods, and rare large-scale floods also play a role. Therefore, the interaction between these floods can markedly impact the formation of riverine plant communities. Such disturbance interactions, caused by the same type of representation, have been overlooked. In this study, we examined a riparian area impacted by Typhoon Hagibis in October 2019, situated along the Akigawa River in Tokyo. Through a comparison of land covers before and after the typhoon, we determined the effects of large-scale flood, and assessed the influence of periodic floods one and two years post-typhoon. Plant community surveys were conducted in both post-typhoon periods, investigating the relationship between community structure using both α- and β-diversity and disturbance interaction regimes. One year postdisturbance, the plant communities strongly reflected the varying intensities of the large-scale flood disturbance. However, two years postdisturbance, community structures exhibited changes, likely influenced by interactions with periodic disturbances. The results of this study suggest that interactions with the same type of disturbance may enhance community diversity.

## Introduction

Disturbance is a fundamental ecological event that reshapes ecosystem structures, profoundly influencing biological communities and their diversity^1–3^. Disturbance effects are characterized by their type, frequency, scale, and intensity, and extensive research has explored the impact of individual disturbances on biological communities^4–6^. However, disturbances do not act in isolation. Ecosystems reflect the impacts of past disturbances, and new disturbances can indirectly interact with previous disturbances^4,5,7^. In recent years, disturbances interactions, a previously overlooked phenomenon, have become a topic of interest in ecology and ecosystem management^4,5,7–9^.

Interactions among disturbances arise when a prior disturbance either amplifies or suppresses the impact of a subsequent disturbance, yielding community changes distinct from those caused by a single disturbance^4,5,8,10^. For instance, Simler et al. (2018) demonstrated that the interaction between forest fires and beetle outbreaks might impact species survival differently compared with each disturbance occurring independently. Shinoda and Akasaka (2020) found that the interplay between forest gap formation and ungulate grazing influences germination from seed banks in forest understories, diverging from the outcomes of isolated events. Although the outcomes depend on disturbance type and scale, it is widely accepted that disturbance interactions introduce complex changes, thereby reshaping community structures^4,5,11^.

Biological communities in riparian areas are shaped under the strong influence of disturbances, with flooding being the primary driver. These areas owe their structure and diversity to river channel erosion and deposition processes resulting from frequent flood events^12–15^. In monsoon Asia, including Japan, riparian areas experience periodic seasonal floods. Consequently, many resident species have adapted to these recurrent floods, leading to the formation of specialized communities^1,3,16^. Among the various river-dwelling organisms, plants are particularly influenced by floods, which directly affect their dispersion, establishment, and growth^17,18^. Thus, the resulting plant communities strongly reflect the impacts of flooding disturbances^16,19,20^.

Floods, a significant disturbance factors in riparian areas, can be classified into at least two types: seasonal periodic floods, and rare large-scale floods that occur infrequently. As seasonal periodic floods are relatively uniform in magnitude, species under these conditions adapt, resulting in minimal community structure changes^1,5^. In contrast, rare large-scale floods, such as those caused by typhoons, can dramatically alter both community structures and entire ecosystems functions^1,21–23^. According to the framework of disturbances interaction which a prior disturbance either amplifies or suppresses the impact of a subsequent disturbance^4,5,8,10^, the large-scale disturbances can act as legacies, interacting with subsequent periodic disturbances, either enhancing or suppressing their effects, potentially shaping community structures differently compared with periodic disturbances alone^4,13,24^. The phenomenon of the same type of disturbances interacting may be an aspect that has been overlooked in previous research on disturbance interactions.

To investigate how the interaction between periodic disturbances and rare large-scale disturbances influences riparian plant communities, we focused on the riparian area affected by Typhoon Hagibis in October 2019. This typhoon brought record postwar hourly rainfall to several parts of Eastern Japan, triggering extensive river embankment failures and large-scale flooding (Ministry of Land, Infrastructure, Transport, and Tourism, Hokuriku Regional Development Bureau 2020). In the subsequent years (2020 and 2021), there were no similar flood events, and periodic rainy seasons were observed (Appendix S1 Fig. S1). Consequently, the formed riparian plant communities were likely influenced by the interaction between the large-scale flooding disturbance as a legacy and the periodic disturbances.

In this study we aimed to test how the large-scale floods caused by Typhoon Hagibis, in combination with subsequent periodic disturbances, influenced the formation of riparian plant communities.

To test this, we evaluated the impact of flood disturbances and plant communities along the Akigawa River in Akiruno City, Tokyo, Japan, which has suffered extensive typhoon damage. Initially, we analyzed land cover changes before and after the typhoon using aerial photographs to assess the large-scale disturbance’s impact on the riparian area. We then categorized disturbance intensities of the large-scale disturbance based on land cover changes. Subsequently, we examined land cover changes in 2020 and 2021, categorizing disturbance intensities of periodic disturbances based on land cover changes in each category of large-scale disturbance. This allowed use to classify disturbance interactions as high intensity in large-scale disturbance and low intensity in periodic disturbance. Additionally, we investigated plant communities in each disturbance intensity one and two years after the typhoon. We explored the relationship between these interaction types and plant communities, interpreting the effects of the disturbance interactions on plant communities. Our findings highlight the processes of plant community formation under the influence of disturbance interactions.

## Results

### Effects of disturbances for three land categories

The results of the generalized linear model (GLM) for the land areas showed significant differences among the years for both bare ground and grassland (Appendix Table 1). Accordingly, we conducted Tukey’s multiple comparison test before and after the 2019 typhoon, between after the 2019 typhoon and 2020, and between 2020 and 2021 for each combination of these land cover types. The changes in the areas of each land cover type from before the 2019 typhoon to 2021 are presented in Table 1. There were no significant differences in the bare ground area for each time series combination (*p* < 0.05) (Table 1). Conversely, grassland area exhibited a significant decrease between before and after the 2019 typhoon (*p* < 0.0001; Table 1) but significantly increased from after the 2019 typhoon to 2020 (*p* < 0.0001; Table 1). Forest areas remained relatively stable, with no significant differences observed during the study period (Table 1).

**Table 1:** Summary of land cover areas in the survey units (n = 52).

Land cover type	Bare ground area (Mean ± S.D.)	*p*	Grassland area (Mean ± S.D.)	*p*	Forest (woodland) area (Mean ± S.D.)	*p*
2019 before typhoon	2095.77 ± 2100.30		5956.29 ± 4507.74		392.034 ± 700.41	
		0.695		< 0.0001		–
2019 after typhoon	2732.52 ± 2893.98		569.69 ± 1378.67		462.48 ± 947.74	
		0.14		< 0.0001		–
2020	3987.77 ± 3282.91		5826.64 ± 3808.96		386.03 ± 795.07	
		0.996		0.85		–
2021	4107.33 ± 3457.90		5275.33 ± 3393.68		652.37 ± 1285.50	

The p-value represents the result of comparing the land cover of the upper year with that of the lower year using a Tukey’s pair comparisons test based on results on generalized linear model (GLM). If *p* values were “–”, that indicates significant differences among years in GLM were not detected.

If the *p* values of Tukey’s all-pair comparisons test were less than 0.0001, we did not the specific values.

### Classification of survey units based on large-scale disturbance

Among the 52 survey units, 17 were categorized as low intensity with large-scale disturbance, 17 were classified as medium intensity, and 18 were assigned to the high-intensity class (Table 2). In both 2020 and 2021, the largest bare ground area was observed in the high-intensity class, whereas the smallest was in the low-intensity class (Table 2). In contrast, for grassland, the low-intensity class exhibited the largest area, with the high-intensity class having the smallest area in both 2020 and 2021 (Table 2). Regarding forest areas, the medium class had the largest area in 2020, whereas the low-intensity class exhibited the largest area in 2021 (Table 2). Within each class, there were no significant differences in areas of bare ground, grassland, and forest between 2020 to 2021 excluded the low-intensity class in forest (Table 2).

**Table 2:** Summary of land cover areas in each large disturbance classes in the survey units.

Year	Class	Bareground area (Mean ± S.D.)	*p*	Grassland area (Mean ± S.D.)	*p*	Forest (woodland) area (Mean ± S.D.)	*p*
2020	Low (n = 17)	2982.12 ± 2697.51	–	6619.71 ± 4180.61	–	346.00 ± 910.05	0.049
	Med (n = 17)	3975.35 ± 3245.95	–	5979.41 ± 3326.02	–	513.88 ± 902.59	–
	High (n = 18)	4949.28 ± 3684.73	–	4933.33 ± 3894.39	–	303.11 ± 570.05	–
2021	Low (n = 17)	2995.24 ± 2769.27		6050.88 ± 3849.71		720.71 ± 1788.15	
	Med (n = 17)	4036.18 ± 3577.56		5534.65 ± 3506.26		609.18 ± 1043.25	
	High (n = 18)	5224.83 ± 3747.44		4297.94 ± 2711.41		628.61 ± 957.44	

The p-value represents the result of comparing the values for each class of the land cover between 2020 and 2021 using Tukey’s all-pair comparisons test based on results on generalized linear model (GLM). If *p* values were “–”, that indicates significant differences between years in GLM were not detected.

Based on classification by large-scale disturbance, line transects were established in 17, 17, and 18 locations for the low-, medium-, and high-intensity disturbance classes, respectively. During the vegetation survey, 85 and 74 species were recorded in 2020 and 2021, respectively (Appendix Table S2). In total, 105 species were identified, including morpho species (Appendix Table S2). Among these, 47 species were annual, whereas 42 were perennial (Appendix Table S2).

### Effects of large- and small- scale disturbances for plant species diversity

**Fig. 1:**
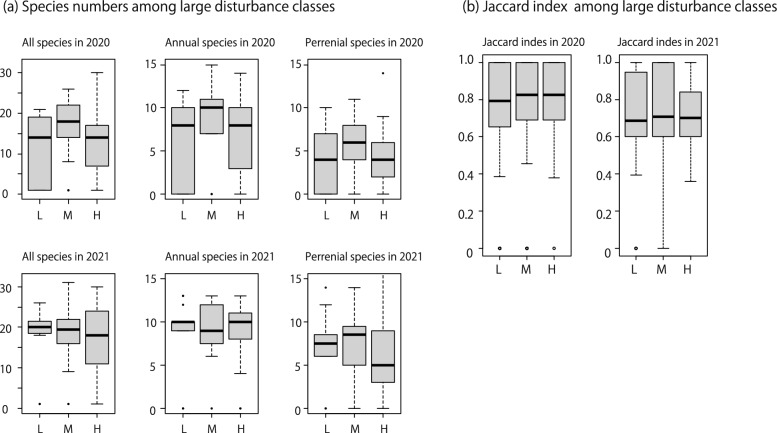
(**a**) Plant species numbers for each lifeform in each large disturbance class in both 2020 and 2021. (**b**) Jaccard index in each large disturbance class in both 2020 and 2021. H: indicates large- intensity flooding, M: indicated medium- intensity flooding, and L: indicated low- intensity flooding, respectively.

Common trends were observed in the three large-scale disturbance classes regarding total, annual, and perennial species numbers in 2020 (Table 3; Fig. 1a). Specifically, the medium class exhibited the highest values in all cases (Table 3; Fig. 1a). In 2021, there were no discernible trends among classes for total, annual, and perennial species numbers (Table 3; Fig. 1a). The Jaccard index showed no particular trends among classes in both 2020 and 2021 (Table 4; Fig. 1b).

**Table 3:** Plant species numbers categorized by lifeform and large-scale disturbance classes.

Year	Class	Total species number (Mean ± S.D.)	*p*	Annual plants number (Mean ± S.D.)	*p*	Perennial plants number (Mean ± S.D.)	*p*
2020	All (n = 52)	13.41 ± 8.29		6.94 ± 4.69		4.47 ± 3.32	
	Low (n = 17)	12.06 ± 8.22	0.005	5.94 ± 4.59	0.0047	3.76 ± 3.07	0.087
	Med (n = 17)	15.65 ± 8.29	–	8.41 ± 4.68	–	5.29 ± 3.48	–
	High (n = 18)	12.53 ± 8.38	0.016	6.47 ± 4.72	0.042	4.35 ± 3.41	0.25
2021	All (n = 52)	16.96 ± 8.33		8.52 ± 4.19		6.46 ± 4.18	
	Low (n = 17)	17.19 ± 8.33	0.7	8.44 ± 4.37	0.72	6.75 ± 3.97	0.69
	Med (n = 17)	17.75 ± 8.08	–	8.81 ± 4.05	–	7.13 ± 4.26	–
	High (n = 18)	16.06 ± 8.93	0.23	8.33 ± 4.34	0.63	5.61 ± 4.37	0.093

The *p*-value indicates the result of the *Wald test*, with the medium class used as the reference standard.

**Table 4:** Jaccard index reflecting plants surveys in each plot in different large disturbance classes.

Year		Jaccard index (Mean ± S.D.)	*p*
2020	All	0.79 ± 0.23	
	Low	0.76 ± 0.26	0.34
	Mid	0.79 ± 0.27	–
	High	0.80 ± 0.17	0.12
2021	All	0.73 ± 0.21	
	Low	0.71 ± 0.22	0.19
	Mid	0.74 ± 0.22	–
	High	0.73 ± 0.17	0.49

The *p*-value indicates the result of the *Wald test*, with the medium class used as the reference standard.

**Fig. 2:**
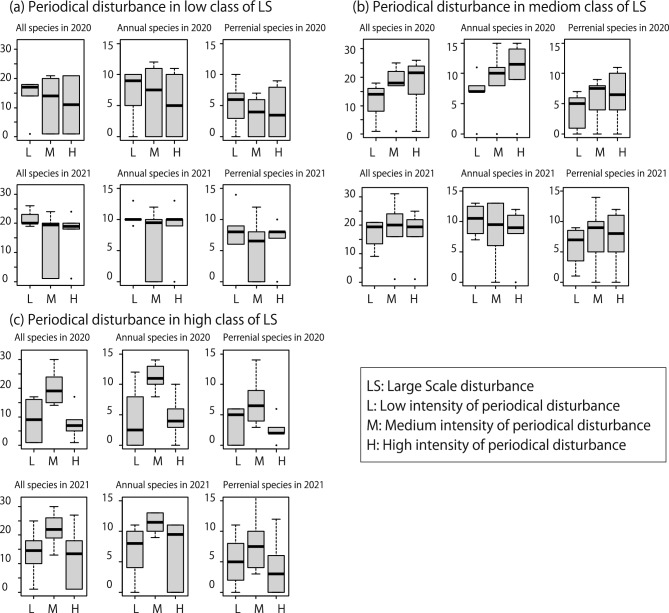
(**a**) Plant species numbers for each lifeform in each periodic disturbance class within the low-intensity class of large scale disturbance. The same values within (**b**) the medium-intensity class of large scale disturbance and (**c**) the high-intensity class of large scale disturbance in both 2020 and 2021. H: indicates large- intensity flooding, M: indicated medium- intensity flooding, and L: indicated low- intensity flooding, respectively.

**Fig. 3:**
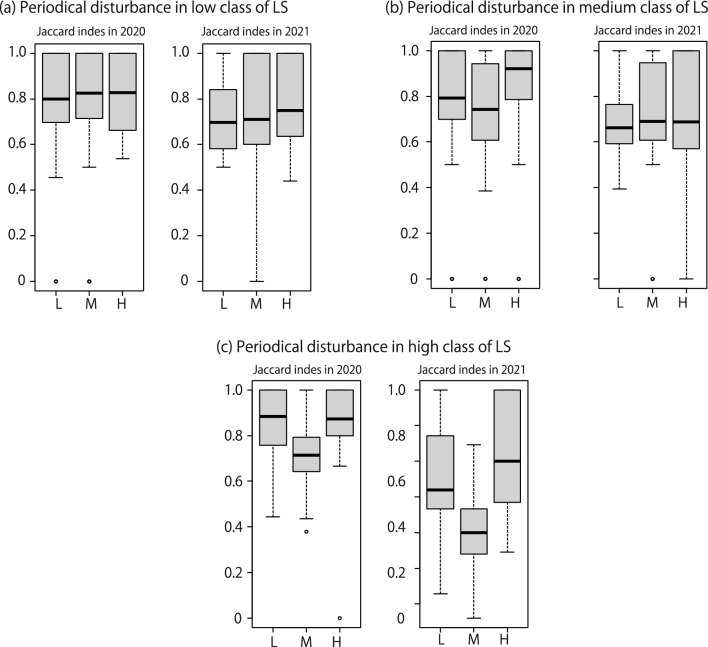
(**a**) Jaccard index in each periodic disturbance class within the low-intensity class of large-scale disturbance. The same values within (**b**) the medium-intensity class of large-scale disturbance and (**c**) the high-intensity class of large-scale disturbance in 2020 and 2021. H: indicates large- intensity flooding, M: indicated medium- intensity flooding, and L: indicated low- intensity flooding, respectively.

Regarding the low-intensity class of large-scale disturbance, both the total species numbers and annual species numbers in 2021 with low-intensity periodic disturbance were significantly greater than those of the medium class (Table 5; Fig. 2a). Regarding the medium-intensity class of large-scale disturbance, there were no significant differences in species numbers among periodic disturbance classes (Table 5; Fig. 2b). Regarding the high-intensity class of large-scale disturbance, total, annual, and perennial species numbers in the medium-intensity periodic disturbance class, in both 2020 and 2021, were significantly higher than those in other classes (Table 5; Fig. 2c). Regarding the Jaccard index, there were no significant differences in both low and medium classes in the high-intensity class (Table 6; Fig. 3a,b), but the index was significantly low in medium class in periodic disturbance in high class in the high-intensity class classes in both 2020 and 2021 (Table 6; Fig. 3c).

**Table 5:** Plant species numbers for each lifeform.

Large-scale disturbance class	Year	Periodical disturbance class	Total species number (Mean ± S.D.)	*p*	Annual plants number (Mean ± S.D.)	*p*	Perennial plants number (Mean ± S.D.)	*p*
Low	2020	Low (n = 5)	13.6 ± 7.23	0.41	6.8 ± 4.32	0.76	5.2 ± 3.83	0.18
		Med (n = 6)	11.83 ± 9.43	–	6.33 ± 5.32	–	3.5 ± 3.22	–
		High (n = 6)	11.0 ± 9.03	0.67	5.17 ± 5.08	0.4	4.00 ± 3.85	0.66
	2021	Low (n = 5)	21.6 ± 2.88	0.004	10.4 ± 1.51	0.044	8.6 ± 3.29	0.053
		Med (n = 6)	14.17 ± 10.34	–	6.83 ± 5.38	–	5.5 ± 4.72	–
		High (n = 6)	16.4 ± 8.91	0.34	8.4 ± 4.93	0.34	6.6 ± 3.85	0.46
Medium	2020	Low (n = 5)	11.4 ± 6.91	0.018	6.6 ± 4.04	0.16	3.80 ± 3.11	0.11
		Med (n = 6)	16.83 ± 8.33	–	9.0 ± 4.98	–	6.0 ± 3.41	–
		High (n = 6)	18 ± 9.27	0.63	10.17 ± 5.49	0.51	6.33 ± 4.13	0.82
	2021	Low (n = 5)	18.2 ± 5.36	0.61	10.2 ± 2.39	0.37	6.6 ± 3.36	0.29
		Med (n = 6)	18 ± 11.16	–	8.2 ± 5.45	–	7.6 ± 5.32	–
		High (n = 6)	17.17 ± 8.52	0.54	8.17 ± 4.26	0.84	7.33 ± 4.41	0.75
Large	2020	Low (n = 6)	8.83 ± 6.97	< 0.0001	4.17 ± 4.83	< 0.0001	3.67 ± 2.88	0.011
		Med (n = 6)	20.17 ± 6.27	–	11.17 ± 2.23	–	7.17 ± 4.26	–
		High (n = 6)	7.80 ± 5.93	< 0.0001	4.60 ± 3.71	0.0002	2.60 ± 2.19	0.0013
	2021	Low (n = 6)	13.83 ± 8.33	0.0009	6.83 ± 4.12	0.011	5.17 ± 4.02	0.058
		Med (n = 6)	22.00 ± 5.97	–	11.33 ± 1.63	–	8.00 ± 4.77	–
		High (n = 6)	12.33 ± 10.07	< 0.0001	6.83 ± 5.42	0.01	4.00 ± 4.52	0.0056

Values represent subdivisions of large-scale disturbance classes into periodic disturbance classes. The *p*-value indicates the result of the *Wald test*, with the medium class used as the reference standard.

If the *p* values of *Wald test* was less than 0.0001, we did not the specific values.

**Table 6:** Jaccard index among plant surveys in each plot.

Large-scale disturbance class	Year	Periodical disturbance class	Jaccard index (Mean ± S.D.)	*p*
Low	2020	Low	0.76 ± 0.31	0.85
		Med	0.77 ± 0.30	–
		High	0.83 ± 0.17	0.31
	2021	Low	0.72 ± 0.18	0.74
		Med	0.74 ± 0.26	–
		High	0.77 ± 0.19	0.49
Medium	2020	Low	0.78 ± 0.24	0.19
		Med	0.72 ± 0.26	–
		High	0.81 ± 0.299	0.12
	2021	Low	0.71 ± 0.17	0.87
		Med	0.70 ± 0.25	–
		High	0.75 ± 0.26	0.41
Large	2020	Low	0.86 ± 0.16	< 0.0001
		Med	0.71 ± 0.14	–
		High	0.86 ± 0.17	< 0.0001
	2021	Low	0.76 ± 0.16	< 0.0001
		Med	0.61 ± 0.11	–
		High	0.83 ± 0.17	< 0.0001

Values represent subdivisions of the large-scale disturbance class into periodic disturbance classes. The *p*-value indicates the result of the *Wald test*, with the medium class used as the reference standard.

## Discussion

This study assessed the interplay of disturbances in a riparian area affected by both large-scale flooding from Typhoon Hagibis and periodic flooding due to seasonal rains on plant communities. One year after the typhoon, the plant community exhibited unimodal biodiversity patterns, indicating the effects of the large-scale disturbance. Notably, the interaction between high-intensity large-scale flooding and periodic flooding resulted in unimodal biodiversity patterns at the local scale, persisting for one and two years after the typhoon. This suggests that interactions between the same type of disturbances; large-scale and periodic flooding influence local plant community formation.

### Effects of large-scale flooding on the riparian area

Comparing conditions before and after Typhoon Hagibis in 2019, a significant decrease in natural terrestrial area was observed. Specifically, the grassland area experienced a drastic reduction, whereas the bare ground area increased gradually. This shift can be attributed to the impact of flooding, which led to the removal of vegetation and the deposition of sediment. By 2020, one year post-typhoon, grassland areas had significantly increased compared with immediately after the 2019 typhoon. However, no significant differences were observed in that between 2020 and 2021, which only experienced periodic disturbances. These findings suggest that the typhoon constituted a large-scale disturbance leading to land cover changes on a scale not observed with periodic disturbances. Flooding is a major factor contributing to the creation of bare ground areas in riparian zones and strongly influences plant communities^3,16^. Therefore, Typhoon Hagibis likely had a lasting impact on the plant communities across the study area.

The survey units were classified into three categories based on the impact of Typhoon Hagibis as determined by changes in the natural terrestrial area. Setting thresholds for variable categorization can be subjective, necessitating validation^25^. Comparing the number of plant species among classes in 2020, which is likely to retain the impact of the typhoon, it was evident that the class with medium disturbance intensity exhibited the highest diversity. This aligns with the intermediate disturbance hypothesis^26^, which suggests that the highest diversity occurs under moderate disturbances. Although this pattern was not mirrored in the Jaccard index, these findings indicate that the classification of disturbance intensity used in this study was appropriate, at least concerning its impact on plant communities in the study area. In this study, the Jaccard index represented the diversity of local communities within a class. The absence of differences among classes suggests that, after large-scale flooding, the habitat quality in each class became considerably homogeneous. This result is consistent with the observation that species diversity varied among classes.

### Plant communities change after large-scale flooding

Although the number of plant species in 2020 was the highest in the medium disturbance intensity class of large-scale disturbance, this pattern was not evident in 2021. However, in 2021, the average species numbers in all cases increased compared with those in 2020. Moreover, in 2020, annual species numbers exhibited a distinct unimodal pattern, which was not observed in 2021. Notably, the average number of annual species was highly similar between the medium class in 2020 and all classes in 2021. These results suggest that annual species were added to both low- and high-intensity classes in 2021. Typically, annual species favor disturbed sites as pioneer species, whereas perennial species tend to prefer areas with relatively milder disturbances in riparian areas^3,16^. However, a previous study suggested that the response to disturbance can vary even within annual plants^27^. Therefore, in 2020, following the typhoon, annual species that favor strong disturbances may have invaded areas with high disturbance intensity, whereas species that favor relatively weak disturbances may have invaded areas with low disturbance intensity. This led to medium-intensity disturbance areas exhibiting the highest species diversity, consistent with the intermediate disturbance hypothesis. Contrastingly, in 2021, with no large-scale flooding events, habitat heterogeneity caused by large-scale flooding was less pronounced, leading to the loss of the unimodal pattern in annual species diversity.

Perennial species exhibited a relatively weak but consistent unimodal pattern over 2 years. Although the pattern remained, the number of species increased in all classes. Perennial species tend to have higher tolerance to disturbances to some degree in riparian areas^16,28^. Therefore, in 2020, perennial species with relatively high disturbance tolerance likely persisted based on the intensity of large-scale flooding. In 2021, species with lower tolerance may have evenly invaded all classes.

### Effects of disturbance interaction for plant communities

Under the high-intensity class of large-scale disturbance, plant species diversity and the Jaccard index showed clear unimodal patterns in both 2020 and 2021 by the subdivided unit which according to the intensity of periodic disturbances. In the high-intensity class of large-scale disturbance units, species with high disturbance preference (i.e., pioneers) may establish themselves preferentially. This process could lead to species composition homogenization, resulting in an increase in the Jaccard index [i.e., a decrease in β-diversity^29^]. However, the actual plant communities showed an increase not only in species numbers (α-diversity) but also in β-diversity. These results suggest that periodic disturbances influenced community formation. One of possible explanation for the increase in both α-diversity and β-diversity is that periodic disturbances suppressed the succession process. Periodic disturbances have a relatively smaller impact range compared with large-scale disturbances. Therefore, they do not uniformly affect all locations. Disturbance intensity can be high in some areas and low in others, creating a gradient^26,30^. Consequently, there might have been differences in the stages of succession within the high-intensity class of large-scale disturbance^31,32^. The coexistence of different succession stages within the same class would lower similarity, leading to increased α-diversity and β-diversity. The phenomenon where a subsequent disturbance alters the effects of a preceding disturbance is frequently noted in research on disturbance interactions^4,5,8,10^.

In the medium- and low-intensity classes of large-scale disturbance, plant species diversity and the Jaccard index did not exhibit patterns similar to those in the high-intensity class. Akin to the high-intensity class of large-scale disturbance, periodic disturbances created a gradient of disturbance impact within these classes. One possible explanation for these results is the total intensity of flooding. In 2020, species numbers in the low-intensity classes of large-scale disturbance were typically lower than those in the high-intensity classes of periodic disturbance. In contrast, species numbers in the medium-intensity classes of large-scale disturbance were generally higher than those in the high-intensity classes of periodic disturbance. Therefore, the combination of low-intensity classes of large-scale disturbance and low-intensity classes of periodic disturbance, as well as the combination of medium-intensity classes of large-scale disturbance and high-intensity classes of periodic disturbance, might have provided a habitat suitable for high species diversity in 2020. In the former case, the area experienced the least disturbance impact across the study area, allowing many species to persist postdisturbance. In the latter case, the area suffered a higher disturbance impact relative to other parts of the study area. This impact likely created a suitable habitat for many plants, including pioneers. However, these patterns were not sustained in 2021, which can also be explained by disturbance interactions. Based on the results for plant species diversity among large-scale disturbance classes, habitat heterogeneity among these classes was less pronounced in 2021. Therefore, the impact of disturbance interaction, specifically the total intensity of flooding between large-scale and periodic flooding, appeared to have diminished.

### Limitation and future challenges

This study successfully detected the phenomenon in which interactions between disturbances of the same type. However, this study has some limitations. The first is the spatial scale limitation. In this study, disturbance intensity was defined based on changes in land cover. Based on the results of the plant species number, this classification seems to have some validity, at least within the survey area, and the resulting findings have a certain degree of reliability. However, the surveyed area covered only 5 km of the Akigawa River, which is a small part of the entire river system. Land-cover conditions likely differ among parts of the river system. For example, bare ground with gravel predominates in the upstream regions and significant urban development in the downstream regions. Therefore, it remains an interesting question for future research whether the interactions among disturbances of varying intensities affect and how to evaluate these for the entire river ecosystem. The second is data limitation. Ideally, to detect the impact of disturbance interactions, it might be appropriate to include an interaction term for large-scale and small-scale disturbances in the statistical model. However, owing to data constraints, this study conducted a two-step analysis to evaluate the impact of small-scale disturbances within classes defined by the intensity of large-scale disturbances, without directly detecting interactions. Also, we used only indicators related to the plant community that could be addressed were the species number and Jaccard index, which means that species richness could not be evaluated due to the limitation of the survey efforts. Moreover, how the disturbance interactions examined in this study affect taxonomic groups other than plants remains an important question. These limitations can be resolved if large-scale projects allow for the collection of more widespread data.

## Conclusion

This study suggests that disturbance interactions, which have previously been discussed in the context of different types of disturbances^4,5,8,10^, may also occur in disturbances of the same type but varying intensities. Such disturbance interactions, caused by the same type of representation, have been overlooked. Such disturbance interactions, caused by the same type of representation, have been overlooked. Disturbances, such as the floods addressed in this study, often include both small-scale events and rare, large-scale ones. Also, discussions in previous studies have typically revolved around whether an initial disturbance suppresses or promotes subsequent disturbances^4,5,7,8,10^. In contrast, the present study suggests that disturbance interactions have the potential to enhance community diversity, particularly in the context of large-scale disturbances. By delving deeper into the processes of biodiversity and community assembly through the exploration of disturbance interactions, we aim to advance our understanding of the intricate relationship between disturbances and ecosystems.

## Methods

### Study area

**Fig. 4:**
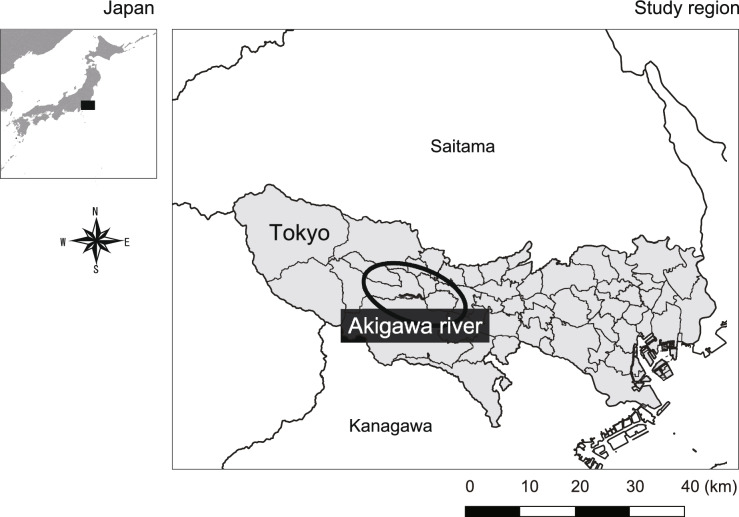
Study area location: Akigawa River.

The survey was conducted in the riparian area of the Akigawa River (Fig. 4), covering a regulated section spanning 33.57 km with a catchment area of 101.9 km^2^, managed by the Tokyo Metropolitan Construction Bureau (Tokyo Metropolitan Construction Bureau, https://www.kensetsu.metro.tokyo.lg.jp/jimusho/nishiken/kanri-ka/4-kasen/kasen.html, accessed on August 30, 2023). This study focused on the area near the confluence with the Tama River, considered a midstream region with relatively stable environmental factors, including riverbed gradient, that can influence plant growth.

Upon reviewing monthly precipitation data from Hachioji City, the closest meteorological observation point to our survey site, it was evident that the monthly rainfall for October 2019 substantially exceeded 600 mm, indicating heavy rainfall associated with a typhoon (Appendix S1 Fig. S1). Additionally, between June and August in 2019, 2020, and 2021, there were smaller peaks in rainfall, suggesting the presence of seasonal rain (Appendix S1 Fig. S1).

### Evaluation of disturbance impact

We assessed disturbance impact by categorizing land cover types within river channels, specifically bare ground, grassland, and forest, before and after the large-scale flooding event in October 2019, as well as one year (2020) and two years (2021) postflooding. Although directly quantifying flood disturbance in riverine areas can be challenging, the presence of bare ground within river channels, a result of flooding effects, serves as a reliable indicator of such disturbance^3,16,33^.

Aerial photographs, captured between June and November 2019, were used to measure areas of land cover, with such photographs of the river preflooding sourced from GSI Maps (https://maps.gsi.go.jp, accessed on August 30, 2023). Although the typhoon passed in October 2019, the aerial photographs showed walking paths and bridges in their pretyphoon state, indicating that the images were captured prior to the typhoon occurring. Additionally, post-typhoon aerial photographs were obtained from GSI Maps, featuring images from October 13, 2019, i.e., shortly after the typhoon (https://maps.gsi.go.jp/development/ichiran.html#t20191012typhoon19_tamagawa_1013do, accessed on August 30, 2023). Aerial photographs taken one year post-typhoon (captured in August 2020) were acquired from NTT InfraNet Corporation’s GEOSPACE aerial photographs (https://www.ntt-geospace.co.jp/geospace/koukuu.html, accessed on August 30, 2023). Finally, aerial photographs taken 2 years after the flood (May 2021) were obtained using a multicopter DJI Phantom 4 Pro (https://www.dji.com/jp/phantom-4-pro, accessed on August 30, 2023), and an orthophoto was created using Pix4Dmapper ver.4.6.4 (https://www.pix4d.com/product/pix4dmapper-photogrammetry-software/, accessed on August 30, 2023). Although the shooting months varied each year, no significant differences in land cover were expected due to the timing of photographs, as they were all taken during the plant growing seasons. The aerial photographs were interpreted, and terrestrial areas within the river channel were classified into three land cover categories: bare ground, grassland, and forest (dominated by woody plants). Polygon data were generated using QGIS 3.10 (https://qgis.org/en/site/, accessed on August 30, 2023).

**Fig. 5:**
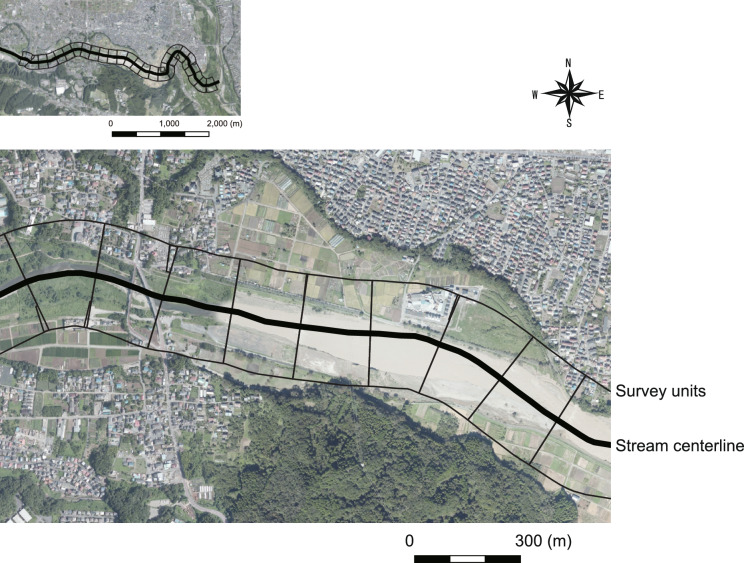
Survey unit description. Each unit spans 300 m in width at approximately 200 m intervals from the stream centerline.

To quantify the changes in land cover as an indicator of disturbance impact, survey units were established. Gregory et al. (1991) recommended that a section length of 10–100 times the channel width is an appropriate spatial scale to assess the impact of natural disturbances on river structure^19^. In this study, the survey area predominantly featured a channel width of 20–30 m. Therefore, buffer polygons measuring 300 m in width were established at approximately 200 m intervals from stream centerline data, sourced from the Ministry of Land, Infrastructure, Transport, and Tourism’s National Land Numerical Information (https://nlftp.mlit.go.jp/ksj/gml/datalist/KsjTmplt-W05.html, accessed on August 30, 2023). Each buffer polygon was subdivided into north and south sections (Fig. 5), resulting in a total of 52 units for analysis. These divided buffers were used as units for land cover and subsequent field surveys (as described later).

The bare ground, grassland, and forest areas in each unit were derived from the created polygons. To quantify changes in land cover over time, we calculated the areas of each category in each unit, both before and after the typhoon in 2019, 2020, and 2021. Areas of land cover that were water or other types, such as agricultural land, artificial land, and constructions, were excluded from the analysis.

### Classification of large-scale flooding impact

We categorized survey units into three classes based on the disturbance intensity caused by large-scale flooding: high, medium, and low intensity. This classification was based on a comparison of land cover data from before and after the 2019 typhoon, serving as a direct reflection of the large-scale flooding event’s impact. Classification criteria were based on the loss of natural terrestrial area, encompassing the total area of the three land categories we established. If > 50% of the land area was lost due to the flooding, the unit was designated as high intensity. For land area loss between 25% and < 50%, units were assigned medium intensity. If land area loss was < 25%, the unit was classified as low intensity.

### Classification of periodic flooding impact

Further classification of survey units into three classes, within each large-scale flooding impact category, was based on the disturbance intensity caused by periodic flooding. This classification involved the use of land cover data from 2020 and 2021, comparing land cover changes between years. As no large-scale flooding occurred in these years, land cover changes were indicative of the effects of periodic disturbances. Classification criteria were chosen according to changes in natural terrestrial area, considering the total area of the three land categories for this period. Differences in the natural terrestrial area of each unit between 2020 and 2021 were calculated and ranked within each large-scale flooding intensity category. Units with the largest third of differences were designated as high intensity, the next third as medium intensity, and the smallest third as low intensity. Consequently, each of the three large-scale flooding intensity categories had three periodic flooding intensity categories within them.

### Vegetation survey

Vegetation surveys were conducted in each survey unit from early August to early September in 2020 and from late July to late August in 2021. The survey involved establishing a 20 m line transect from the boundary between the river flow and the terrestrial area in each unit, and recording the plant species along the transect. In each survey transect, we recorded the occurrence of annual plant species, perennial plant species, and other plant species, including woody species. The surveys focused on the presence of species. For each unit in each year, we counted the total species number, annual species number, and perennial species number as an α-diversity index. We also calculated the Jaccard index based on the composition of plant species among each unit as a β-diversity index. The Jaccard index, *J*, was calculated as follows:

1 $$J = \frac{{S_{AB} }}{{S_{A} + S_{B} - S_{AB} }},$$

where S_A_ and S_B_ represent the species number in site A and B, respectively, and S_AB_ indicates the common species number between site A and B. The Jaccard index is bounded between 0 and 1, with 0 indicating no shared species between the two sites, and 1 indicating identical species composition. We calculated the Jaccard index for transect pairs in the same class of large-scale disturbance and in the same class of periodic disturbance within the large-scale disturbance class. The former reflected β-diversity between large-scale disturbances, whereas the latter reflected β-diversity between the periodic disturbance classes within the same class of large-scale disturbance.

### Statistical analysis

To evaluate the impact on natural terrestrial area by both large-scale and periodic disturbances across the entire study area, we used a generalized linear model (GLM) with Gaussian distributions (identity link) and a Tukey’s multiple comparison test to compare the areas of the three types of land cover among study periods. The explanatory variables were years; these were 2019 before the typhoon, 2019 after the typhoon, 2020, and 2021. If significant differences among these years were detected, we compared land cover areas between before and after the 2019 typhoon, between after the 2019 typhoon and 2020, and between 2020 and 2021 for each combination in each land cover type. To evaluate the influence of periodic disturbances on terrestrial area in each land cover type in each class of large-scale flooding impact, we compared the areas of the three land cover types between 2020 and 2021 using the same approach.

To assess the influence of large-scale disturbances on plant communities in the riparian area, we examined the total species number, annual species number, and perennial species number in each unit using generalized linear model (GLM) with Poisson distributions (log link) and a *Wald test*. The explanatory variable was the large-scale disturbance category (low, medium, and high) in the survey units. Given the categorical nature of the explanatory variable, the medium level served as the reference standard. Similarly, we assessed the Jaccard index in each unit using a GLM with Gaussian distributions (identity link) and a *Wald test*. The explanatory variable was the large-scale disturbance category in the survey units. Additionally, to evaluate the influence of plant communities resulting from the interaction between large-scale and periodic disturbances, we conducted a similar analysis, using periodic flooding classes in each large-scale disturbance class. For example, in the high-intensity class of large-scale disturbance, three classes of periodic disturbance were present. Thus, each periodic disturbances class was defined as follows: (1) high–high, (2) high–medium, and (3) high–low intensities (referring to the large-scale disturbance–periodic disturbances combination). We analyzed both species numbers and Jaccard index using GLMs based on periodic flooding classes as the explanatory variable in the survey units.

All statistical analysis were performed using the R statistical package (version 4.4.2; R development core Team, https://www.r-project.org/, accessed at 30, November, 2024).

## Supplementary Information

Supplementary Information 1.

Supplementary Information 2.

Supplementary Information 3.

Supplementary Information 4.

## Data Availability

The datasets used and/or analyzed during the current study are available as supplemental material.
