# Supplementary material for: Multiple floods interactions shape riparian plant communities and diversity
Source: Sci Rep. 2025 Jul 2;15:23567. doi: 10.1038/s41598-025-05938-6 (PMC12222478; doi:10.1038/s41598-025-05938-6)
Supplement: Supplementary file 1 — Supplementary Information 1. [file 41598_2025_5938_MOESM1_ESM.pdf]

Appendix Table S1. Land area among survey years. The p-value refers to the result of the Wald test when using the 2019 before typhoon as a reference standard.

Year		Coefficients (Estimated ± S.E.)	*p*
Bare ground	2019		0.27
	(after typhoon)	636.8±585.4	
	2020	1892.0±585.4	0.001
	2021	2011.6±585.4	0.001
	Intercept	2095.8±413.2	
Grassland	2019		<0.001
	(after typhoon)	-5386.6±681.1	
	2020	-129.7±681.1	0.84
	2021	681.0±681.1	0.32
	Intercept	5956.3±481.6	
Forest	2019		0.71
	(after typhoon)	70.4±187.9	
	2020	-6.0±187.9	0.97
	2021	260.3±187.9	0.17
	Intercept	392.0±132.9	

If the *p* values was less than 0.001, we did not the specific values
