# Supplementary material for: Multiple floods interactions shape riparian plant communities and diversity
Source: Sci Rep. 2025 Jul 2;15:23567. doi: 10.1038/s41598-025-05938-6 (PMC12222478; doi:10.1038/s41598-025-05938-6)
Supplement: Supplementary file 2 — Supplementary Information 2. [file 41598_2025_5938_MOESM2_ESM.pdf]

Appendix Table S2. Plant species list for the vegetation survey.

Scientific Name	Family	Life type	2020	2021
Aceraceae sp.	Aceraceae	-	1	0
Achyranthes bidentata Blume var. fauriei (H.L?v. et Vaniot)	Amaranthaceae	perennial	1	1
Aeschynomene indica L.	Fabaceae	annual	1	0
Agrimonia pilosa Ledeb.	Rosaceae	perennial	1	0
Agrimonia pilosa Ledeb. var. japonica (Miq.) Nakai	Rosaceae	perennial	1	0
Allium tuberosum Rottler ex Spreng.	Amaryllidaceae	perennial	0	1
Alnus sieboldiana Matsum.	Betulaceae	woody	1	1
Ambrosia trifida L.	Asteraceae	annual	1	1
Ampelopsis glandulosa (Wall.) Momiy. var. heterophylla (Thunb.) Momiy.	Vitaceae	woody	1	1
Amphicarpaea edgeworthii Benth.	Fabaceae	annual	0	1
Artemisia indica Willd. var. maximowiczii (Nakai) H.Hara	Asteraceae	perennial	1	1
Asteraceae sp.	Asteraceae	-	1	0
Atocion armeria (L.) Raf.	Caryophyllaceae	annual	1	1
Bidens biternata (Lour.) Merr. et Sherff	Asteraceae	annual	1	0
Bidens frondosa L.	Asteraceae	annual	1	1
Bidens pilosa L. var. pilosa	Asteraceae	annual	1	1
Boehmeria nivea (L.) Gaudich. var. concolor Makino f. nipononivea (Koidz.) Kitam. ex H.Ohba	Urticaceae	perennial	1	1
Capsella bursa-pastoris (L.) Medik.	Brassicaceae	annual	1	1
Capsella bursa-pastoris (L.) Medik. var. triangularis Grunner	Brassicaceae	annual	1	1
Causonis japonica	Vitaceae	perennial	1	1
Celosia argentea L.	Amaranthaceae	annual	1	0
Chamaecrista nomame (Makino) H.Ohashi	Fabaceae	annual	0	1
Chenopodium album L.	Amaranthaceae	annual	1	1
Cirsium japonicum	Asteraceae	perennial	0	1
Clematis terniflora DC.	Ranunculaceae	woody	1	1
Commelina communis L.	Commelinaceae	annual	1	1
Convolvulaceae sp.	Convolvulaceae	perennial	1	0
Convolvulaceae sp.	Convolvulaceae	perennial	1	0
Coreopsis tinctoria Nutt.	Asteraceae	annual	1	1
Cosmos bipinnatus	Asteraceae	annual	0	1
Cosmos sulphureus Cav.	Asteraceae	annual	1	1
Cyperus microiria Steud.	Cyperaceae	annual	1	1
Dennstaedtia hirsuta (Sw.) Mett.	Dennstaedtiaceae	annual	0	1
Dysphania ambrosioides (L.) Mosyakin et Clemants	Amaranthaceae	annual	1	1
Eclipta alba (L.) Hassk.	Asteraceae	annual	1	0
Equisetum arvense L.	Equisetaceae	perennial	1	1
Erigeron annuus	Asteraceae	annual	1	1
Erigeron bonariensis L.	Asteraceae	annual	1	1
Erigeron canadensis L.	Asteraceae	annual	1	1
Erigeron sumatrensis Retz.	Asteraceae	annual	1	1
Euphorbia maculata L.	Euphorbiaceae	annual	1	1
Fallopia japonica (Houtt.) Ronse Decr. var. japonica	Polygonaceae	perennial	1	1
Geum japonicum Thunb.	Rosaceae	perennial	1	0
Glycine max (L.) Merr. subsp. soja (Siebold et Zucc.) H.Ohashi	Fabaceae	annual	1	1
Helianthus tuberosus L.	Asteraceae	perennial	1	1
Humulus scandens (Lour.) Merr.	Cannabaceae	annual	1	1
Hydrocotyle maritima Honda	Araliaceae	perennial	1	1
Hypochaeris radicata L.	Asteraceae	perennial	0	1
Indigofera pseudotinctoria Matsum.	Fabaceae	woody	1	1
Lactuca indica L.	Asteraceae	annual	1	1
Liriope muscari (Decne.) L.H.Bailey	Asparagaceae	perennial	1	1
Lycium chinense Mill.	Solanaceae	woody	1	1
Lysimachia clethroides Duby	Primulaceae	perennial	1	0
Macleaya cordata (Willd.) R.Br.	Papaveraceae	perennial	1	1
Mazus pumilus (Burm.f.) Steenis	Mazaceae	annual	0	1
Miscanthus sacchariflorus (Maxim.) Benth. et Hook.f. ex Franch.	Poaceae	perennial	1	0
Nasturtium officinale R.Br.	Brassicaceae	perennial	1	1
Oenanthe javanica (Blume) DC.	Apiaceae	perennial	0	1
Oenothera biennis L.	Onagraceae	annual	1	1
Oenothera laciniata Hill	Onagraceae	annual	1	1
Oenothera rosea L'H?r. ex Aiton	Onagraceae	perennial	1	1
Oldenlandia brachypoda DC.	Rubiaceae	annual	1	0
Oxalis corniculata L.	Oxalidaceae	perennial	1	1
Oxalis corniculata L. f. villosa (M.Bieb.) Goiran	Oxalidaceae	perennial	1	1
Paederia foetida L.	Rubiaceae	perennial	1	1
Paulownia tomentosa (Thunb.) Steud.	Paulowniaceae	woody	1	1

Appendix Table S2. Continued.

Scientific Name	Family	Life type	2020	2021
Perilla frutescens (L.) Britton var. crispa (Benth.) W.Deane	Lamiaceae	annual	1	1
Persicaria longiseta (Bruijn) Kitag.	Polygonaceae	annual	1	1
Persicaria senticosa (Meisn.) H.Gross	Polygonaceae	annual	1	1
Persicaria thunbergii (Siebold et Zucc.) H.Gross	Polygonaceae	annual	1	0
Persicaria thunbergii (Siebold et Zucc.) H.Gross var. thunbergii	Polygonaceae	annual	1	0
Phragmites australis	Poaceae	perennial	0	1
Phragmites japonicus Steud.	Poaceae	perennial	1	1
Phytolacca americana L.	Phytolaccaceae	perennial	1	1
Plantago asiatica L.	Plantaginaceae	perennial	1	1
Plantago asiatica L. var. densiuscula Pilg.	Plantaginaceae	perennial	1	1
Plantago japonica Franch. et Sav.	Plantaginaceae	perennial	1	0
Poaceae sp.	Poaceae	-	1	0
Poaceae sp.	Poaceae	-	1	0
Poaceae sp.	Poaceae	perennial	1	0
Potentilla freyniana Bornm.	Rosaceae	perennial	1	0
Pueraria lobata (Willd.) Ohwi subsp. lobata	Fabaceae	perennial	1	1
Robinia pseudoacacia L.	Fabaceae	woody	1	1
Rosa multiflora Thunb.	Rosaceae	woody	1	1
Rumex obtusifolius L.	Polygonaceae	perennial	1	1
Salicaceae sp.	Salicaceae	woody	1	0
Salix miyabeana	Salicaceae	woody	0	1
Setaria viridis (L.) P.Beauv.	Poaceae	annual	1	1
Setaria viridis (L.) P.Beauv. var. minor (Thunb.) Ohwi	Poaceae	annual	1	1
Sicyos angulatus L.	Cucurbitaceae	annual	1	1
Solanum carolinense L.	Solanaceae	perennial	0	1
Solanum nigrum L.	Solanaceae	annual	1	0
Solidago altissima L.	Asteraceae	perennial	1	1
Sonchus asper (L.) Hill	Asteraceae	annual	1	0
Thalictrum aquilegiifolium L.	Ranunculaceae	perennial	1	0
Thalictrum aquilegiifolium L. var. intermedium Nakai	Ranunculaceae	perennial	1	0
Torenia fournieri Linden ex E.Fourn.	Linderniaceae	annual	1	0
Trichosanthes cucumeroides (Ser.) Maxim. ex Franch. et Sav.	Cucurbitaceae	annual	0	1
Trifolium pratense L.	Fabaceae	perennial	1	1
Trifolium repens L.	Fabaceae	perennial	1	1
Verbena bonariensis L.	Verbenaceae	annual	0	1
Veronica anagallis-aquatica L.	Plantaginaceae	perennial	1	1
Veronica persica Poir.	Plantaginaceae	annual	1	0
Vigna angularis	Fabaceae	annual	0	1
Vitis ficifolia Bunge	Vitaceae	woody	1	1
