# Supplementary figures and images for: Multiple floods interactions shape riparian plant communities and diversity
Source: Sci Rep. 2025 Jul 2;15:23567. doi: 10.1038/s41598-025-05938-6 (PMC12222478; doi:10.1038/s41598-025-05938-6)

(mm / month)

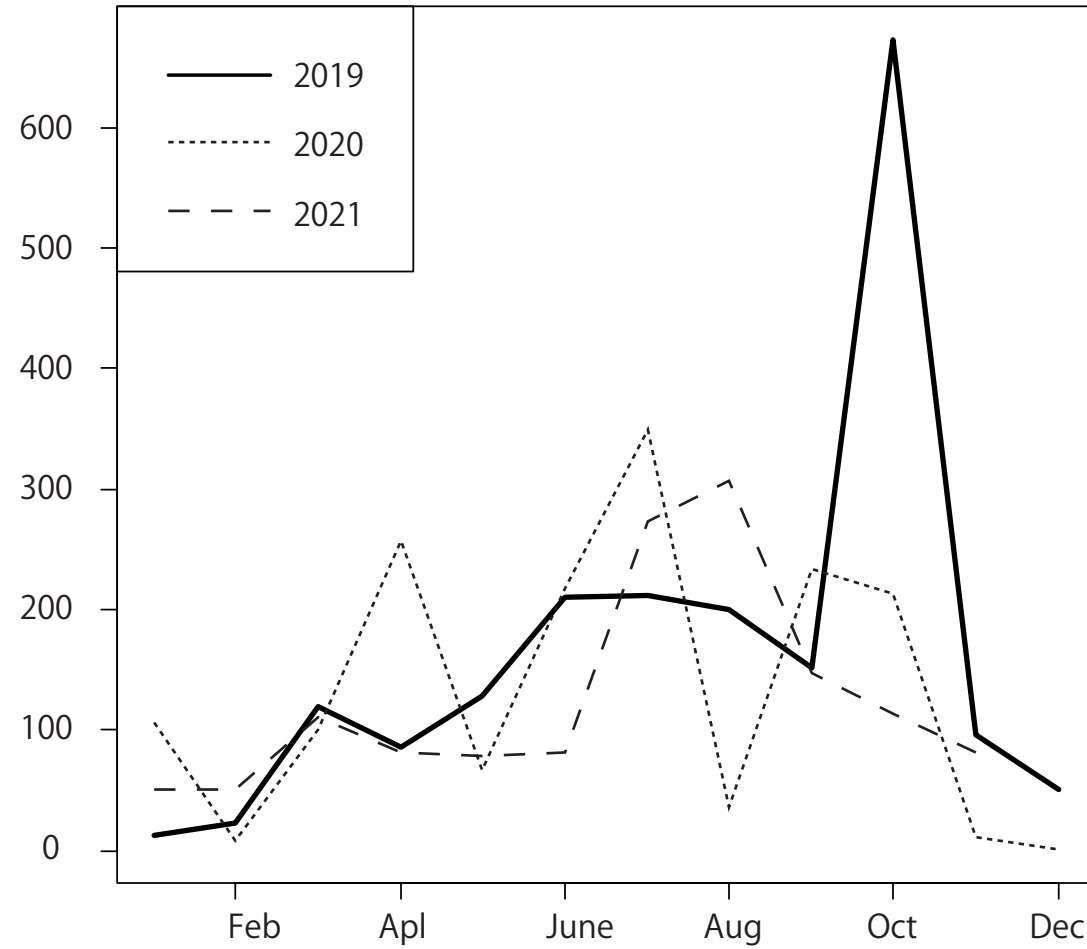

Supplement: Supplementary file 3 — Supplementary Information 3. [file 41598_2025_5938_MOESM3_ESM.pdf]
